# Altering rRNA 2’O-methylation pattern during neuronal differentiation is regulated by FMRP

**DOI:** 10.1080/15476286.2025.2563986

**Published:** 2025-10-03

**Authors:** Michelle Ninochka D’Souza, Naveen Kumar Chandappa Gowda, Nivedita Hariharan, Syed Wasifa Qadri, Dasaradhi Palakodeti, Ravi S Muddashetty

**Affiliations:** a Centre for Brain Research, Indian Institute of Science, Bangalore, India; b Department of Chemical Biology Approaches for Stem Cells and Therapeutics (ICB), Institute for Stem Cell Science and Regenerative Medicine, GKVK Campus, Bangalore, India; c Centre for Advance Research and Excellence in Autism and Developmental Disorders (CAREADD), St. John’s Research Institute, St. John’s National Academy of Health Science, Bangalore, India; d Manipal Academy of Higher Education (MAHE), Manipal, India

**Keywords:** FMRP, ribosome, snoRNA, epitranscriptome, Ribosomal RNA

## Abstract

The Fragile X Messenger Ribonucleoprotein (FMRP) is a selective RNA-binding protein that localizes to the cytoplasm and the nucleus. The loss of FMRP results in Fragile X Syndrome (FXS), an autism spectrum disorder. FMRP interacts with ribosomes and regulates the translation of mRNAs essential for neuronal development and synaptic plasticity. However, the biochemical nature of this translation regulation is unknown. Here, we report that a potential feature of FMRP-mediated translation regulation during neuronal differentiation is the modulation of 2’-O-methylation of ribosomal RNA. 2’O-methylation, facilitated by C/D box snoRNAs in the nucleus, is a major epitranscriptome mark on rRNA, essential for ribosome assembly and function. We found that FMRP influences a distinct rRNA 2’O-Methylation pattern across neuronal differentiation. We show that in H9 ESCs, FMRP interacts with a selected set of C/D box snoRNA in the nucleus, resulting in the generation of ribosomes with a distinct pattern of rRNA 2’O-Methylation. This epitranscriptome pattern on rRNA undergoes a significant change during the differentiation of ESCs to neuronal precursors and cortical neurons. ESCs exhibit substantial levels of hypomethylated residues on rRNA, which progressively decrease in neuronal precursors and post-mitotic cortical neurons. This reduction correlates with changes in global protein synthesis across different stages of differentiation. Importantly, this stepwise change in the 2’O-methylation pattern during neuronal differentiation is altered in the absence of FMRP, which could impact neuronal development and contribute to dysregulated protein synthesis observed in Fragile X Syndrome.

## Introduction

Dynamic change in the protein repertoire mediated by translation regulation is a critical determinant of Embryonic stem cell (ESCs) maintenance and differentiation [1,2]. Several factors, including non-coding RNAs, RNA-binding proteins, and epitranscriptomic modifications such as mRNA m6A modifications, are shown to alter translation rates, thereby regulating the protein repertoire of ESCs [3]. Recent work has shown that rRNA modifications that vary between cell types could potentially regulate translation by modulating the interaction between mRNA and ribosomes. However, the role of epitranscriptomic modification at the rRNA level and its contribution to ESC differentiation is less explored [4]. 2’O-methylations are one of the major epitranscriptomic marks found on rRNA. In humans, C/D box small nucleolar RNAs (snoRNAs) guide the addition of 2’O-methylation on the ribose sugar of rRNA, and this process can occur either co-transcriptionally or post-transcriptionally [5]. rRNA methylation is important for ribosome biogenesis as it helps in the folding of rRNA and assembly of the ribosomes [6]. The idea of ribosomes being structurally and functionally uniform entities has been seriously challenged in recent years, and the idea of ribosome heterogeneity is gaining wide acceptance [7–9]. Ribosome heterogeneity can be attributed to the content and the modifications of both proteins and rRNA. Though the change in the protein composition of ribosomes was shown to have a regulatory role in translation, the consequence of rRNA-based ribosome heterogeneity and its role in translation regulation is only recently gaining recognition [10].

In our previous work, we showed that the differential pattern of rRNA 2’O-methylation in Shef4 hESCs was contributed by a large extent of hypomethylated residues [11]. Further, our work also demonstrated that Fragile X Messenger Ribonucleo-binding protein (FMRP), an RNA-binding protein, modulates this rRNA methylation at several specific sites, generating a differential 2’O-methylation pattern [11]. Consequently, the differential 2’O-methylation pattern on the ribosome assists in the binding of FMRP, which plays an important role in the regulation of protein synthesis [4,11]. FMRP-mediated translation regulation is critical for brain development and functioning [12–14]. Consequently, the loss of FMRP results in a severe form of autism spectrum disorder called Fragile X Syndrome, which is primarily characterized by intellectual disability [15].

In this study, we investigated the dynamic changes in rRNA 2′-O-methylation during the maintenance of human embryonic stem cells (hESCs) and their differentiation into neural lineages, providing a framework for future studies on its functional significance. Our results reveal that neural stem cells (NSCs) exhibit higher translation rates than hESCs, suggesting that changes in cell state could be associated with shifts in global protein synthesis. Specifically, we found that rRNA hypomethylation is broadly associated with reduced translation activity, as observed in wild-type (WT) hESCs, whereas hypermethylation correlates with increased translation, as seen in WT NSCs. This trend also holds true in FMR1 knockout (KO) hESCs.

We captured significant alterations in the 2′-O-methylation landscape of rRNA during differentiation, with hESCs displaying the highest number of hypomethylated sites compared to NSCs and neurons. Importantly, differential methylation patterns were evident in both monosomes and polysomes, suggesting that methylation status may directly influence the ultimate pool to which a ribosome belongs. Furthermore, we show that the transition from ESCs to NSCs involves regulated changes in rRNA methylation mediated by specific small nucleolar RNAs (snoRNAs) in coordination with Fragile X Mental Retardation Protein (FMRP). Together, this study provides insights into 2’O-methylation-dependent translation regulation mediated by FMRP and its importance in the differentiation of ESCs to neural lineages.

## Results

### rRNA is hypomethylated in ESCs, and methylation increases as they differentiate into NSCs and neurons

**Figure 1.**
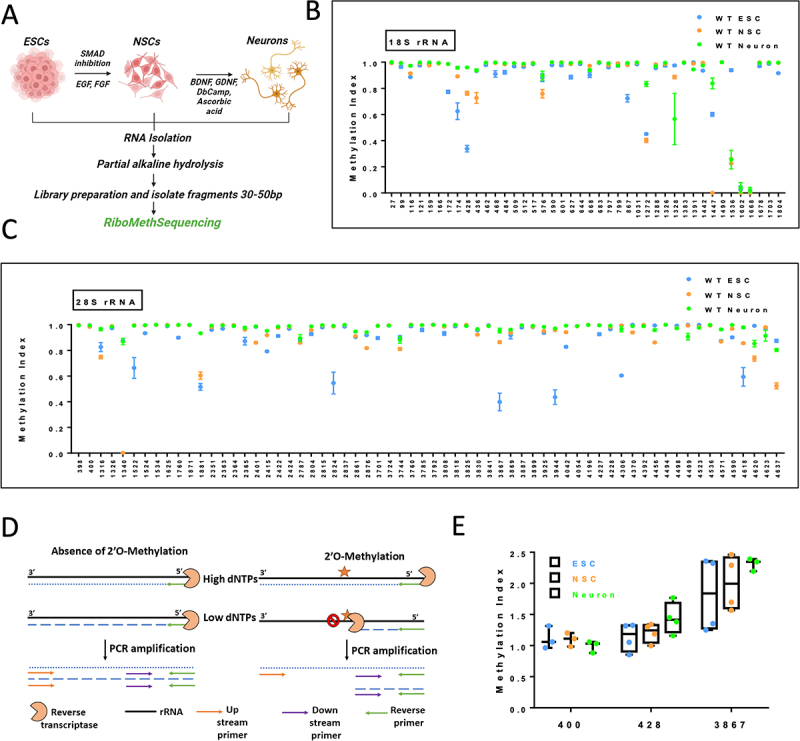
Changes in 2’O-Methylation pattern of rRNA along neuronal differentiation of H9 ESCs.

We sought to investigate the changes in 2’O-methylation marks on ribosomal RNA during ESC differentiation to neuronal precursors and mature neurons. For this purpose, H9 ESCs were differentiated into Neural Stem Cells (NSCs) and finally into forebrain glutamatergic neurons through the inhibition of the SMAD signalling pathway (Figure S1A-C). RNA from ESCs, NSCs, and differentiated neurons was subjected to RiboMeth-Seq (RMS) to estimate the changes in the 2’O-methylation patterns across 18S and 28S rRNA as described previously [15,16]. In brief, 2 micrograms of total RNA were subjected to controlled alkaline hydrolysis followed by library preparation and sequencing (Figure 1(A)). The extent of 2’O-methylation of specific sites of the 18S and 28S rRNA is represented as methylation indices (MI). MI = 1 indicates complete methylation at a particular site, while MI = 0.1 indicates a methylation of only 10% of the rRNA population at a particular site. RiboMeth scores for ESCs, NSCs, and neurons indicate differential patterns of 2’O-methylation in 18S and 28S rRNA across these three stages of neural differentiation (Figure 1(B,C)). Our RiboMeth-Seq captured a total of 103 differentially methylated sites with 39 residues in 18S rRNA and 64 residues in 28S rRNA, respectively (Figure 1(B,C)). Further, we captured two distinct patterns in our RMS score: a) Among the three differentiation stages, ESC rRNA displayed the highest number of partially 2’O-methylated sites (4 sites in ESC 18S rRNA and 8 sites in ESC 28S rRNA). This indicates that ESCs contain a maximum number of ribosomes having hypomethylated residues, and b) the number of 2’O-hypomethylated residues decreases as the ESCs differentiate to NSCs and reduces even further as the NSCs undergo transition to post-mitotic neurons (2sites in NSC 18S rRNA and 2 sites in NSC 28S rRNA). (Figure 1(B,C) and Figure S1D and S1E). Detailed information on sites that show a significant increase in methylation across ESC to neurons is provided in Tables 1 and 2. Conversely, a few sites in 18S and 28S rRNA show a significant shift to hypomethylation in NSCs and neurons compared to ESC (indicated in Tables 3 and 4), which is an opposite trend observed in the majority of the sites mentioned earlier. The pattern of 2’O-methylation obtained was distinct among H9 ESCs and the differentiated NSCs and neurons. However, the hypomethylated residues in H9 ESC were the same as those captured in our previous study using an alternate ESC line, Shef4 [11].

A. Schematic for RiboMeth-Sequencing from H9 ESCs, H9 NSCs, and H9 neurons. B. Methylation Index of the sites on 18S rRNA in H9 ESCs, H9 NSCs, and H9 neurons. The x-axis represents the methylation position on 18S rRNA, and the y-axis represents the Methylation Index. Data is represented as Mean ± SEM, *n* = 3 for ESCs, *n* = 4 for NSCs, and *n* = 4 for neurons. C. Methylation Index of the sites on 28S rRNA in H9 ESCs, H9 NSCs, and H9 neurons. The x-axis represents the methylation position on 28S rRNA, and the y-axis represents the Methylation Index. Data is represented as Mean ± SEM, *n* = 3 for ESCs, *n* = 4 for NSCs, and *n* = 4 for neurons. D. Schematic of PCR-based RTL-P assay used to identify 2’O-methylation status of individual sites. E. Box plot representing the changes in the Methylation Index for sites 400, 428, and 3867 between H9 ESCs, H9 NSCs, and H9 neurons measured through RTL-P. The box extends from the 25th to the 75th percentile, with the middlemost line representing the median of the dataset. Whiskers range from minimum to maximum data points. *n* = 3–4 independent experiments. One-way ANOVA was performed for a given site, and the multiple comparison test was performed with its ESC, NSC, and neuron state values.

**Table 1.** Sites in 18S rRNA show a methylation shift between states of neuronal differentiation. One-way ANOVA was calculated for each site with Tukey’s MCT.

18S rRNA							
nucleotide	ESC WT	NSC WT	Neuron WT	ANOVA_p_value	Tukey’s MCT p value		
					ESC vs NSC	ESC vs NEURON	NSC vs NEURON
27	0.993126	0.993779	1	0.429	0.993	0.492	0.507
99	0.964139	0.994675	0.9924365	0.00293	0.0038	0.006	0.927
116	0.886409	0.913716	0.9727528	< 0.0001	0.0511	< 0.0001	0.0004
121	0.975219	0.992299	0.9968943	0.00215	0.0084	0.002	0.488
159	0.983293	0.975395	0.9979098	< 0.0001	0.0512	0.002	0.0001
166	0.991444	0.990237	0.9942913	0.194	0.853	0.443	0.181
172	0.773057	0.986251	0.9861445	< 0.0001	< 0.0001	< 0.0001	0.999
174	0.62519	0.891424	0.957827	0.000161	0.0007	0.0002	0.282
428	0.338442	0.762446	0.959955	< 0.0001	< 0.0001	< 0.0001	< 0.0001
436	0.932921	0.726662	0.9383833	0.000694	0.0021	0.99	0.0011
462	0.977988	0.995158	0.9960748	0.00913	0.016	0.0122	0.976
468	0.909771	0.994058	0.9978048	0.0013	0.0025	0.0019	0.968
484	0.922278	0.984663	0.991567	0.000655	0.0016	0.0008	0.799
509	0.967931	0.992193	0.9920413	0.00128	0.0021	0.0022	0.999
512	0.964674	0.978501	0.9877608	0.0023	0.0304	0.0018	0.111
517	0.985335	0.994519	0.9905125	0.169	0.148	0.49	0.599
576	0.881844	0.758204	0.8967893	0.0301	0.0777	0.949	0.0352
590	0.977527	0.998987	0.9992265	0.000178	0.0003	0.0003	0.996
601	0.976304	0.998684	0.997167	0.00554	0.0072	0.0106	0.949
627	0.885702	0.984896	0.9938585	< 0.0001	< 0.0001	< 0.0001	0.624
644	0.985892	0.99891	0.9967683	0.000132	0.0001	0.0005	0.395
668	0.902507	0.972097	0.9391228	0.00969	0.0077	0.131	0.143
683	0.994139	0.999611	0.9982948	0.0702	0.0652	0.166	0.773
797	0.960798	0.975845	0.9908723	0.00207	0.0624	0.0016	0.0456
799	0.977258	0.991295	0.973921	0.312	0.501	0.958	0.314
867	0.723714	0.978765	0.9940543	< 0.0001	< 0.0001	< 0.0001	0.677
1031	0.983998	0.996362	0.9975953	< 0.0001	< 0.0001	< 0.0001	0.565
1272	0.450539	0.40198	0.8331725	< 0.0001	0.162	< 0.0001	< 0.0001
1288	0.954468	0.982487	0.9679955	0.0355	0.0298	0.32	0.232
1326	0.974528	0.985004	0.9950905	0.0538	0.345	0.0452	0.32
1328	0.974711	0.886104	0.5649975	0.108	0.881	0.124	0.202
1383	0.994346	0.998894	0.9972805	0.000857	0.0007	0.0098	0.102
1391	0.998099	0.989368	0.9447543	0.000101	0.463	0.0002	0.0003
1442	0.935299	0.994245	0.975143	< 0.0001	< 0.0001	0.0002	0.0133
1447	0.600625	0	0.8380905	< 0.0001	< 0.0001	0.0009	< 0.0001
1490	0.999182	0.99947	0.9994388	0.366	0.381	0.457	0.985
1536	0.938834	0.225773	0.254877	< 0.0001	< 0.0001	0.0001	0.914
1602	0.026374	0	0.0397198	0.591	0.802	0.944	0.571
1668	0.012691	0	0.0212655	0.586	0.83	0.918	0.561
1678	0.970023	0.997875	0.993371	0.00369	0.0039	0.0106	0.701
1703	0.987796	0.997955	0.9969368	0.0273	0.0315	0.0501	0.938
1804	0.915503	0.99427	0.9967138	< 0.0001	< 0.0001	< 0.0001	0.665

**Table 2.** Sites in 28S rRNA show a methylation shift between states of neuronal differentiation. One-way ANOVA was calculated for each site with Tukey’s MCT.

28S rRNA							
nucleotide	ESC WT	NSC WT	Neuron WT	ANOVA_	Tukey’s MCT *p* value		
					ESC vs NSC	ESC vs NEURON	NSC vs NEURON
398	0.998006	0.997673	0.993328	0.0662	0.984	0.101	0.0997
400	0.995383	0.983287	0.993876	0.000101	0.0002	0.64	0.0003
1316	0.825662	0.747368	0.964756	0.000114	0.0585	0.0031	0.0001
1326	0.972769	0.988575	0.984236	0.0138	0.0121	0.0551	0.517
1340	0.873736	0.00154	0.868292	< 0.0001	< 0.0001	0.971	< 0.0001
1522	0.663124	0.994978	0.991648	0.000406	0.0007	0.0007	0.998
1524	0.933567	0.99479	0.995877	< 0.0001	< 0.0001	< 0.0001	0.938
1534	0.998708	0.997734	0.999203	0.136	0.397	0.771	0.123
1625	0.988571	0.998125	0.993423	0.0284	0.0234	0.258	0.231
1760	0.898756	0.996909	0.997488	< 0.0001	< 0.0001	< 0.0001	0.989
1871	0.995832	0.996764	0.999337	0.15	0.85	0.159	0.285
1881	0.515724	0.603932	0.933649	< 0.0001	0.0547	< 0.0001	< 0.0001
2351	0.960067	0.98108	0.992865	0.00011	0.0018	0.0001	0.0287
2363	0.969565	0.997901	0.994424	< 0.0001	< 0.0001	< 0.0001	0.405
2364	0.989246	0.996409	0.99011	0.000843	0.0015	0.792	0.0021
2365	0.871694	0.961157	0.975329	0.00341	0.0089	0.0038	0.772
2401	0.983286	0.85977	0.98724	< 0.0001	< 0.0001	0.842	< 0.0001
2415	0.791581	0.918155	0.952379	< 0.0001	< 0.0001	< 0.0001	0.0003
2422	0.911944	0.975664	0.984815	< 0.0001	< 0.0001	< 0.0001	0.371
2424	0.968366	0.98321	0.988908	< 0.0001	0.0003	< 0.0001	0.0429
2787	0.886671	0.858685	0.894799	0.35	0.558	0.949	0.345
2804	0.92773	0.95782	0.986964	0.000156	0.0093	0.0001	0.0072
2815	0.978796	0.993602	0.996322	0.000541	0.0018	0.0006	0.563
2824	0.545519	0.986259	0.991524	< 0.0001	0.0001	0.0001	0.994
2837	0.984671	0.997805	0.998705	< 0.0001	0.0001	0.0001	0.835
2861	0.903707	0.910871	0.946508	0.0011	0.655	0.0017	0.0033
2876	0.918528	0.81759	0.941158	< 0.0001	< 0.0001	0.0601	< 0.0001
3701	0.895551	0.993059	0.990555	< 0.0001	< 0.0001	< 0.0001	0.912
3724	0.991608	0.995094	0.992761	0.362	0.358	0.881	0.566
3744	0.899003	0.810367	0.881939	0.0243	0.0312	0.818	0.0565
3760	0.987395	1	0.997804	0.0793	0.0797	0.152	0.883
3785	0.959855	0.995976	0.996038	0.00518	0.0081	0.0081	0.999
3792	1	0.99989	0.999024	0.123	0.971	0.163	0.183
3808	0.931588	0.985124	0.993318	0.000398	0.0012	0.0005	0.632
3818	0.987092	0.998393	0.997999	0.00726	0.0102	0.0123	0.988
3825	0.991377	0.998278	0.989947	0.102	0.23	0.927	0.105
3830	0.956589	0.922148	0.96569	< 0.0001	0.0008	0.308	0.0001
3841	0.995722	0.990531	0.98835	0.0444	0.144	0.0387	0.615
3867	0.398386	0.863865	0.954061	< 0.0001	< 0.0001	< 0.0001	0.185
3869	0.917171	0.935685	0.961319	0.143	0.642	0.131	0.394
3887	0.978034	0.995064	0.995065	0.0137	0.0204	0.0204	0.999
3899	0.981098	0.9843	0.990915	0.00142	0.229	0.0014	0.0095
3925	0.936745	0.949838	0.982632	< 0.0001	0.12	0.0001	0.0007
3944	0.43542	0.957662	0.971155	< 0.0001	< 0.0001	< 0.0001	0.922
4042	0.827201	0.940962	0.991644	< 0.0001	< 0.0001	< 0.0001	0.0003
4054	0.987696	0.987296	0.988987	0.907	0.995	0.951	0.904
4196	0.998505	0.997973	0.997221	0.806	0.961	0.796	0.911
4227	0.925469	0.996499	0.994203	< 0.0001	< 0.0001	< 0.0001	0.94
4228	0.964003	0.981646	0.992173	< 0.0001	0.0011	< 0.0001	0.015
4306	0.604514	0.993796	0.965317	< 0.0001	< 0.0001	< 0.0001	0.306
4370	0.998482	0.939396	0.988933	0.00022	0.0004	0.541	0.0007
4392	0.993777	0.998077	0.988699	0.463	0.848	0.796	0.433
4456	0.993466	0.861522	0.958717	< 0.0001	< 0.0001	0.0339	< 0.0001
4494	0.999014	0.999464	0.999342	0.541	0.523	0.7	0.942
4498	0.991991	0.973779	0.971908	0.052	0.0866	0.0595	0.959
4499	0.99781	0.993002	0.907781	0.00678	0.978	0.0134	0.012
4523	0.997831	0.982592	0.975999	0.04	0.137	0.0346	0.589
4536	0.995819	0.993318	0.999119	0.156	0.675	0.516	0.137
4571	0.872787	0.868315	0.963485	< 0.0001	0.897	< 0.0001	< 0.0001
4590	0.901049	0.968143	0.997735	< 0.0001	0.0001	< 0.0001	0.007
4618	0.593665	0.855277	0.979272	0.000166	0.0018	0.0001	0.061
4620	0.991591	0.736001	0.852558	0.000107	0.0001	0.0046	0.0081
4623	0.97455	0.979873	0.913635	0.208	0.99	0.329	0.231
4637	0.874646	0.523633	0.803216	< 0.0001	< 0.0001	0.0504	< 0.0001

**Table 3.** Sites in 18S rRNA indicating nucleotide positions and their respective shift in methylation status.

WT ESC to WT NSC		WT NSC to WT Neuron	
Hyper (21)	Hypo (4)	Hyper (8)	Hypo (2)
99	159	116	1391
116	436	159	1442
121	1447	428	
172	1536	436	
174		576	
428		797	
462		1272	
484		1447	
509			
601			
627			
644			
668			
867			
1031			
1288			
1383			
1442			
1678			
1703			
1804

**Table 4.** Sites in 28S rRNA indicating nucleotide positions and their respective shift in methylation status.

WT ESC to WT NSC		WT NSC to WT Neuron	
Hyper (32)	Hypo (8)	Hyper (22)	Hypo (2)
400	1340	400	2364
1326	2401	1316	4499
1522	2876	1340	
1524	2744	1881	
1625	3830	2351	
1765	4456	2401	
1881	4620	2415	
2351	4637	2424	
2363		2804	
2364		2861	
2365		2876	
2415		3830	
2422		3887	
2424		3925	
2804		4042	
2815		4228	
2824		4370	
2837		4456	
3701		4571	
3784		4590	
3808		4620	
3818		4637	
3867			
3887			
3944			
4042			
4227			
4228			
4306			
4370			
4590			
4618			

Next, we independently validated the changes in 2’O-methylation on specific sites of 18S and 28S rRNA through a qPCR-based tool referred to as RTL-P [17]. Details of the primer design and product amplification have been described in Figure 1(D). We have validated the changes in methylation for three different positions on rRNA- site 428 in 18S rRNA and sites 400 and 3867 in 28S rRNA. Position 391 on 28s rRNA was found to be methylated in all 3 differentiation stages (Figure 1(C)). Sites 428 and 3867 in 18S and 28S rRNA, respectively, show increasing trends of methylation in the NSC and neuronal stages (Figure 1(C)). Using RTL-P, we confirmed the complete methylation of Site 400 across the 3 different cell types and increased methylation of sites 428 and 3867 as the cells differentiated into neurons, validating the RMS data generated by next-generation sequencing (Figure 1(E)). Together, our results show that there is a dynamic change in the 2’O-methylation of rRNA in the differentiation of ESCs, where ESCs have the least amount of 2’O-methylation.

### FMRP affects 2’O-methylation of rRNA majorly in ESCs

Our published work demonstrated a novel role for FMRP in regulating the methylation of 2’O-ribose sugars of specific bases in ESCs [11]. In the current study, we aimed to understand the effect of FMRP on 2’O-methylation of rRNA during the differentiation of ESCs into NSCs and cortical neurons. For this, we used H9 ESC and FMR1 KO H9 ESC lines. The knockout of FMRP was performed through CRISPR-Cas9 deletion of exon 1 of the FMR1 gene (Figure S2A) [18]. FMR1 KO ESCs were characterized for stem cell markers OCT4 and Nanog (Figure S2B and S2E). FMR1 KO ESCs were differentiated into NSCs and neurons as described earlier [19]. Differentiated states were confirmed by the presence of markers such as Nestin and Pax6 in NSCs and MAP2 and VGlut1 in neurons through immunostaining and qPCR of mRNA candidates (Figure S2C, S2D, S2F, and S2G).

**Figure 2.**
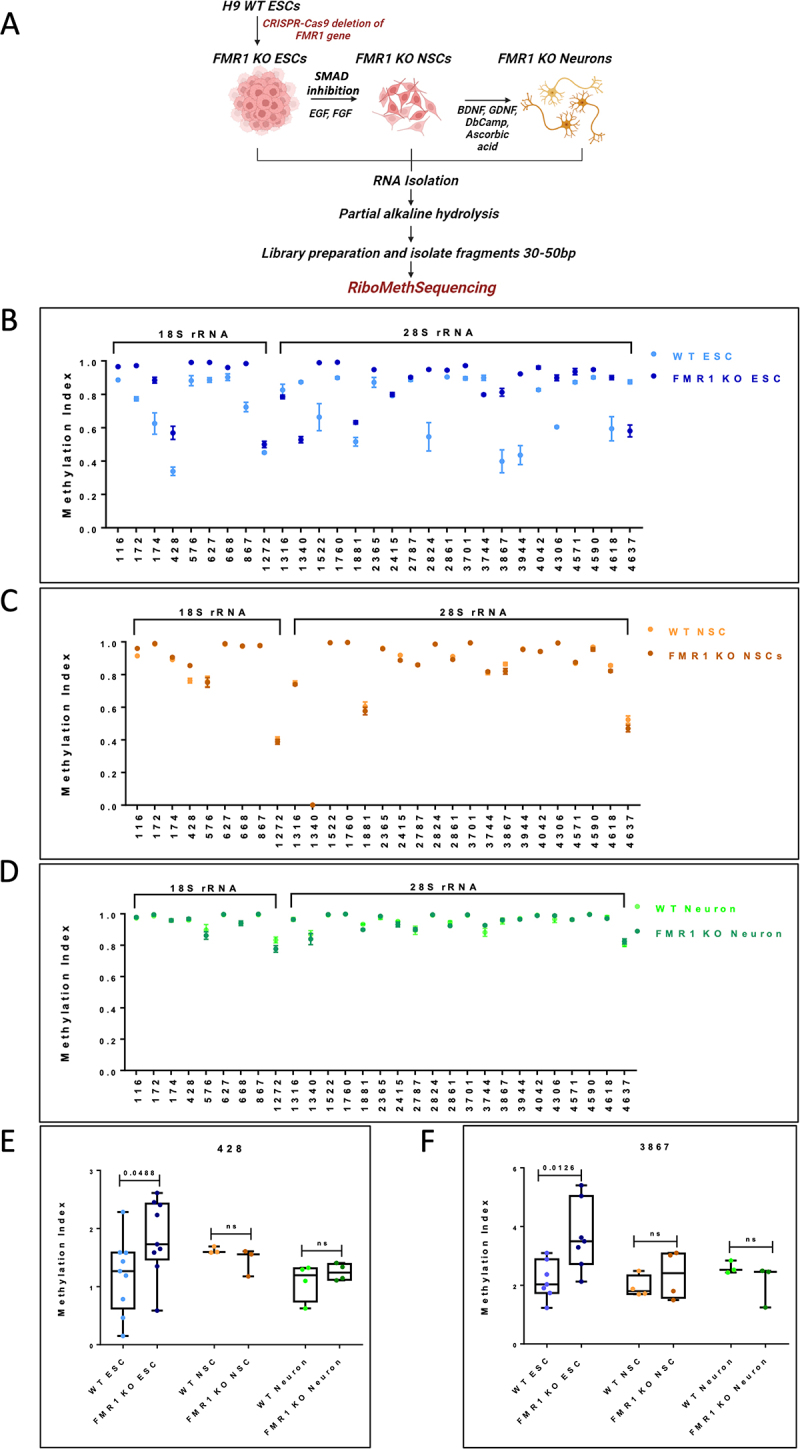
Altered 2’O-Methylation pattern along neuronal differentiation in the absence of FMRP.

To understand how FMRP influences 2’O-methylation levels at each stage of differentiation, we performed RiboMeth-Seq from ESCs, NSCs, and neurons from both WT and FMR1 KO cell lines (Figure 2A). RMS data from FMR1 KO cells indicate maximum hypomethylation of rRNA was in the ESC stage compared to NSCs and neurons, which was similar to our observation in the WT condition. This suggests that the overall trend of increasing 2’O-methylation among ESC, NSC, and mature neurons does not change between WT and FMR1 KO conditions. (Figure 2(B-D), Figure S2H and S2I). However, to study specific changes in 2’O-methylation status due to the loss of FMRP, we selected sites in WT ESC 18S and 28S rRNA that have an MI score less than/equal to 0.9 and examined their MI in the FMR1 KO ESCs (Figure 2(B)). Furthermore, we compared the MI of these sites in WT and FMR1 KO NSCs (Figure 2(C)) and neurons (Figure 2(D)). We observed that the fold difference in 2'O-methylation between the WT and KO conditions of certain sites (e.g. Site 468 in 18S rRNA and Site 2824 in 28S rRNA) drastically reduces from the ESC to NSC to neuronal types (Figure B-D) . Additionally, we plotted a heat map by grouping variable positions and saturated positions across the differentiated cell states and compared them with the FMR1 KO condition (Figure S2J and S2K). We have indicated the sites on 18S and 28S rRNA showing significant changes in 2'O-methylation between WT and FMR1 KO ESCs/NSCs/neurons in Tables 5–7. These results indicate that the number of hypomethylated positions in both 18S and 28S rRNA is reduced over differentiation, suggesting that the effect of FMRP on rRNA 2’O-methylation is maximum in ESCs. To further validate this result, we selected positions 428 from 18S rRNA and 3867 from 28S rRNA. The Methylation Index for these positions was measured by RTL-P in WT and FMR1 KO ESCs, NSCs, and neurons (Figure 2(E,F)). In ESCs, sites 428 and 3867 show a significant increase in methylation status in the absence of FMRP compared to the WT condition. Further validation of the same sites in NSCs and neurons shows no difference in 2’O-methylation between WT and FMR1 KO conditions, suggesting that the role of FMRP in regulating 2’O-methylation is reduced across neuronal differentiation (Figure 2(E,F)).

A. Schematic showing the generation of FMR1 KO H9 ESCs through CRISPR-Cas9. rRNA from FMR1 KO ESCs, FMR1 KO NSCs, and FMR1 KO neurons was further processed for RiboMeth-Seq. B. Methylation indices of selected sites (MI > 0.90) on 18S rRNA and 28S rRNA in H9 WT and FMR1 KO ESCs. The x-axis represents the methylation position on rRNA, and the y-axis represents the Methylation Index. Data represented as Mean ± SEM, *n* = 3. C. Methylation indices of selected sites (from B) on 18S rRNA and 28S rRNA in H9 WT and FMR1 KO NSCs. The x-axis represents the methylation positions on 18S and 28S rRNA, and the y-axis represents the Methylation Index. Data represented as Mean ± SEM, *n* = 4. D. Methylation indices of selected sites (from B) on 18S rRNA and 28S rRNA in H9 WT and FMR1 KO neurons. The x-axis represents the methylation positions on 18S and 28S rRNA, and the y-axis represents the Methylation Index. Data represented as Mean ± SEM, *n* = 4. E. Box plot representing the change in Methylation Index for site 428 between WT and FMR1 KO ESCs, NSCs, and neurons measured through RTL-P. The box extends from the 25th to the 75th percentile, with the middlemost line representing the median of the dataset. Whiskers range from minimum to maximum data points. Unpaired t-test, *n* = 9 for ESCs, *n* = 3 for NSCs and *n* = 4 for neurons. F. Box plot representing the change in Methylation Index for site 3867 between WT and FMR1 KO ESCs, NSCs, and neurons measured through RTL-P. The box extends from the 25th to the 75th percentile, with the middlemost line representing the median of the dataset. Whiskers range from minimum to maximum data points. Unpaired t-test, *n* = 9 for ESCs, *n* = 3 for NSCs, and *n* = 4 for neurons.

**Table 5.** Sites indicating significant difference in 2’O-methylation between WT and FMR1 KO ESC 18S rRNA and 28S rRNA.

		ESC WT	ESC FMR1 KO	P-Value
18S rRNA	116	0.8864	0.9655	0.00017305
	159	0.9833	0.9947	0.0458963
	172	0.7731	0.972	9.2952E–05
	174	0.6252	0.8848	0.0172618
	428	0.3384	0.5689	0.00790355
	468	0.9098	0.9919	0.03013167
	484	0.9223	0.981	0.01861479
	509	0.9679	0.9851	0.04543201
	576	0.8818	0.9908	0.02228417
	627	0.8857	0.9907	0.00191851
	644	0.9859	0.9956	0.00714317
	668	0.9025	0.9606	0.04182707
	867	0.7237	0.9843	0.00078173
	1288	0.9545	0.9685	0.01610787
	1328	0.9747	0.8895	0.00322142
	1383	0.9943	0.998	0.00902449
	1391	0.9981	0.9397	0.01181839
	1442	0.9353	0.9175	0.00311862
	1490	0.9992	0.9981	0.01562398
	1536	0.9388	0.1264	0.00028686
	1804	0.9155	0.9884	8.6571E–05
28S rRNA	398	0.9978	0.9777	0.00698483
	400	0.9954	0.9853	0.00050524
	1326	0.9729	0.9857	0.04449346
	1340	0.8739	0.528	6.4053E–05
	1522	0.6632	0.9893	0.01578187
	1524	0.9336	0.9973	0.00019075
	1625	0.9884	0.9963	0.02518721
	1760	0.8988	0.992	0.00015771
	1881	0.5157	0.6317	0.01323617
	2351	0.9601	0.9977	0.00016025
	2363	0.9695	0.9973	0.00179803
	2364	0.9894	0.9963	0.00201424
	2422	0.912	0.9623	0.00548524
	2424	0.9685	0.9827	0.00087689
	2815	0.979	0.9907	0.04780965
	2824	0.5457	0.9483	0.00912978
	2837	0.9848	0.997	0.00727384
	2861	0.9038	0.9447	0.00043402
	3701	0.8956	0.9717	0.00195189
	3724	0.9916	0.9753	0.04275199
	3744	0.8988	0.7983	0.00356005
	3808	0.9316	0.9833	0.02257632
	3830	0.9567	0.9797	0.01623957
	3867	0.3985	0.8123	0.00467805
	3869	0.9173	0.778	0.03731292
	3887	0.9781	0.9993	0.02039052
	3925	0.9369	0.9753	0.01006758
	3944	0.4353	0.9223	0.0010103
	4042	0.8273	0.9603	0.00028329
	4227	0.9256	0.9933	0.00339875
	4228	0.9638	0.9967	0.00122037
	4306	0.6046	0.8987	8.2345E–05
	4456	0.9934	0.9637	0.0022907
	4499	0.9979	0.9377	0.03272395
	4523	0.9978	0.9903	0.00519714
	4571	0.8729	0.936	0.03591427
	4590	0.901	0.9483	0.00595971
	4618	0.5938	0.9	0.01413858
	4620	0.9915	0.8897	0.00535085
	4637	0.8744	0.5803	0.00145973

**Table 6.** Sites indicating significant difference in 2’O-Methylation between WT and FMR1 KO NSC 18S rRNA and 28S rRNA.

		NSC WT	NSC FMR1 KO	P-Value
18S rRNA	99	0.9947	0.9967	0.01071522
	116	0.9137	0.96	0.0007347
	159	0.9754	0.9814	0.02030539
	166	0.9902	0.9943	0.04352719
	172	0.9863	0.99	0.02795788
	428	0.7624	0.8545	0.00086674
	512	0.9785	0.9842	0.01070892
28S rRNA	398	0.9977	0.9949	0.00781102
	1326	0.9886	0.9921	0.0102442
	2415	0.9182	0.8871	0.00247807
	2861	0.9109	0.8917	0.00568395
	3760	1	0.9976	0.03563853
	4618	0.8553	0.8221	0.01716329

**Table 7.** Sites indicating significant difference in 2’O-Methylation between WT and FMR1 KO Neuron 18S rRNA and 28S rRNA.

		Neuron WT	Neuron FMR1 KO	P-Value
18S rRNA	99	0.9925	0.9978	0.00376031
	172	0.9858	0.995	0.00212042
	436	0.9385	0.9685	0.04156871
	627	0.9938	0.9973	0.02739604
	799	0.974	0.9953	0.00414657
	1804	0.9965	0.9983	0.04479426
28S rRNA	1871	0.9993	0.9968	0.03076601
	1881	0.9338	0.8988	0.0017439
	2861	0.9463	0.9235	0.04291374
	3899	0.991	0.9955	0.00692005
	4054	0.989	0.9773	0.03487105

### rRNA hypermethylation in NSCs is a result of reduced FMRP-snoRNA interaction

**Figure 3.**
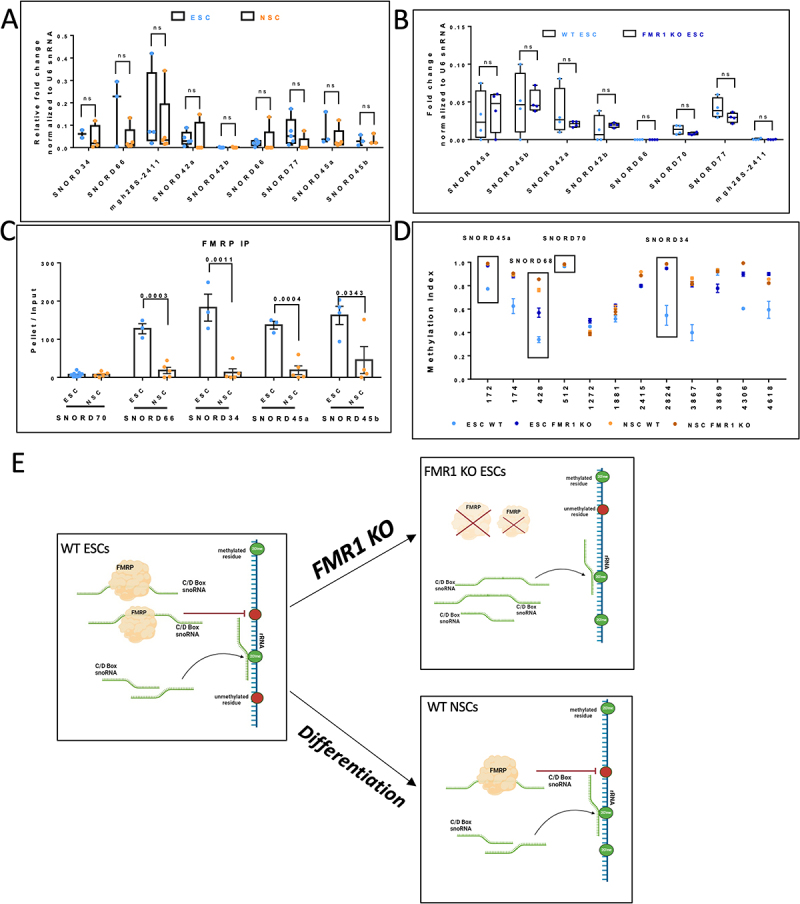
The interaction of FMRP with C/D box snoRNA determines the extent of methylation.

From our results, we observe a trend of hypermethylation in a subset of sites in 18S and 28S rRNA as ESCs differentiate to NSCs. We have previously shown that FMRP interacts with C/D Box snoRNAs and regulates the 2’O-methylation profile of rRNAs in ESCs [11]. Hence, we wanted to investigate the effect of FMRP-snoRNA interaction on 2’O-methylation in the context of neuronal differentiation [11]. To begin with, we examined the relative expression of selected C/D Box snoRNAs in ESCs and NSCs. We chose snoRNA candidates based on the sites that were hypomethylated on 18S and 28S rRNA in WT ESCs. We observed that the steady-state expression of these C/D Box snoRNAs did not significantly alter as ESCs differentiated into NSCs (Figure 3(A)). Further, we observed no significant difference in the levels of the target snoRNAs between WT and FMR1 KO ESCs and NSCs, confirming that FMRP does not affect the steady-state expression of these snoRNAs (Figure 3(B) and Figure S3A).

A. Quantification of selected C/D box snoRNA expression in WT ESC and WT NSC. The graph represents fold change for a given snoRNA normalized to U6 snRNA. The box extends from the 25th to the 75th percentile, with the middlemost line representing the median of the dataset. Whiskers range from minimum to maximum data points. Unpaired t-test, *n* = 3–5. B. Quantification of C/D box snoRNA basal level expression in WT ESC and FMR1 KO ESC. The graph represents fold change for a given snoRNA normalized to U6 snRNA. The box extends from the 25th to the 75th percentile, with the middlemost line representing the median of the dataset. Whiskers range from minimum to maximum data points. Unpaired t-test, *n* = 4. C. Quantification of selected C/D box snoRNA enrichment in FMRP immunoprecipitates from WT ESC and WT NSC. The bar graph represents the pellet/input ratio for a given snoRNA normalized to U6 snRNA. Data represented as Mean ±SEM. Unpaired t-test, *n* = 3–9. D. Methylation indices of the sites on 18S and 28S rRNA in WT H9 ESCs, which get hypermethylated due to a reduction in FMRP-snoRNA interaction (WT NSC) or loss of FMRP (FMR1 KO ESC). Corresponding Methylation indices in the FMR1 KO NSC condition are mentioned. Boxed sites are targeted by FMRP-target snoRNA. The x-axis represents the methylation positions on 18S and 28S rRNA, and the y-axis represents the Methylation Index. *n* = 3. Data represented as Mean ± SEM. E. Model indicating the link between FMRP-snoRNA interaction and methylation pattern. a) WT ESCs- FMRP sequesters C/D Box snoRNAs, leading to rRNA hypomethylation. b) FMR1 KO ESCs- the loss of FMRP increases the availability of free C/D Box snoRNAs, leading to rRNA hypermethylation, and c) WT NSCs – reduced binding of FMRP to C/D Box snoRNAs leads to rRNA hypermethylation on selected sites, resulting in a similar change in Methylation Index as the FMR1 KO ESC condition.

Since we did not capture any alterations in the levels of snoRNAs along differentiation or in the FMR1 KO condition, we investigated whether the altered 2’O-methylation pattern between WT ESCs and WT NSCs could be due to differences in FMRP-snoRNA interactions. Our previous work indicated that FMRP binds to several C/D box snoRNAs in ESCs as well as in NSCs [11]. However, the extent of FMRP-snoRNA interaction between these two cell states was not known. To test this, we performed an FMRP-immunoprecipitation from ESCs and NSCs and quantified the copy number of specific snoRNAs that were bound to FMRP in these two systems through qPCR (Figure 3(C)). We observed that the extent of binding of selected snoRNAs to FMRP is significantly reduced in WT NSCs in comparison to WT ESCs (Figure 3(C)).

We mapped the FMRP-bound snoRNAs to their respective target sites on rRNA to examine the changes in 2’O-methylation between WT ESCs, FMR1 KO ESCs, WT NSCs, and FMR1 KO NSCs (Figure 3(D)). We observe that the sites corresponding to FMRP-bound snoRNAs shift from a hypomethylation to a hypermethylation state between WT and FMR1 KO ESCs (Figure 3(D)). Similarly, we see a similar shift from hypomethylation to hypermethylation when we compare WT ESCs and WT NSCs for these sites (Figure 3(D)). Thus, FMRP has a strong affinity to selected C/D Box snoRNAs in the WT ESCs, which results in the hypomethylation of the sites targeted by these snoRNAs (Figure 3(E)). This interaction is lost in FMR1 KO ESCs or is reduced in the case of WT NSCs, both of which result in hypermethylation of the sites targeted by the FMRP-bound snoRNAs (Figure 3(E) and Table 8).

**Table 8.** Sites indicating significant increase in 2’O-Methylation in FMR1 KO ESC and WT NSC compared to WT ESC 18S rRNA and 28S rRNA.

		WT ESC	FMR1 KO ESC	WT NSC	p value (WT-KO)	*p* value (WT-WT)
18S rRNA	116	0.8864	0.9655	0.9137	0.00017305	0.03219763
	172	0.7731	0.972	0.9863	9.2952E–05	4.823E–06
	174	0.6252	0.8848	0.8914	0.0172618	0.00422618
	428	0.3384	0.5689	0.7624	0.00790355	1.8906E–05
	468	0.9098	0.9919	0.9941	0.03013167	0.00997906
	484	0.9223	0.981	0.9847	0.01861479	0.00577054
	509	0.9679	0.9851	0.9922	0.04543201	0.00322236
	627	0.8857	0.9907	0.9849	0.00191851	0.00056564
	644	0.9859	0.9956	0.9989	0.00714317	0.00054306
	668	0.9025	0.9606	0.9721	0.04182707	0.00880117
	867	0.7237	0.9843	0.9788	0.00078173	0.00012891
	1288	0.9545	0.9685	0.9825	0.01610787	8.1222E–05
	1383	0.9943	0.998	0.9989	0.00902449	0.00045899
	1804	0.9155	0.9884	0.9943	8.6571E–05	4.385E–06
28S rRNA	1326	0.9729	0.9857	0.9886	0.04449346	0.00041523
	1522	0.6632	0.9893	0.995	0.01578187	0.00449013
	1524	0.9336	0.9973	0.9948	0.00019075	2.445E–05
	1625	0.9884	0.9963	0.9981	0.02518721	0.00272706
	1760	0.8988	0.992	0.9969	0.00015771	1.1515E–05
	2351	0.9601	0.9977	0.9811	0.00016025	0.00122109
	2363	0.9695	0.9973	0.9979	0.00179803	0.00028791
	2364	0.9894	0.9963	0.9964	0.00201424	0.00039702
	2422	0.912	0.9623	0.9757	0.00548524	0.00044248
	2424	0.9685	0.9827	0.9832	0.00087689	0.00032957
	2815	0.979	0.9907	0.9936	0.04780965	0.00807616
	2824	0.5457	0.9483	0.9863	0.00912978	0.00160361
	2837	0.9848	0.997	0.9978	0.00727384	0.00136586
	3701	0.8956	0.9717	0.9931	0.00195189	7.937E–05
	3808	0.9316	0.9833	0.9851	0.02257632	0.0066252
	3867	0.3985	0.8123	0.8639	0.00467805	0.00051967
	3887	0.9781	0.9993	0.9951	0.02039052	0.01709752
	3944	0.4353	0.9223	0.9577	0.0010103	0.00010914
	4042	0.8273	0.9603	0.941	0.00028329	5.5552E–05
	4227	0.9256	0.9933	0.9965	0.00339875	0.00041095
	4228	0.9638	0.9967	0.9816	0.00122037	0.00435809
	4306	0.6046	0.8987	0.9938	8.2345E–05	2.549E–09
	4590	0.901	0.9483	0.9681	0.00595971	0.00069823
	4618	0.5938	0.9	0.8553	0.01413858	0.00792276

### Loss of FMRP results in pronounced protein synthesis defects in ESCs

**Figure 4.**
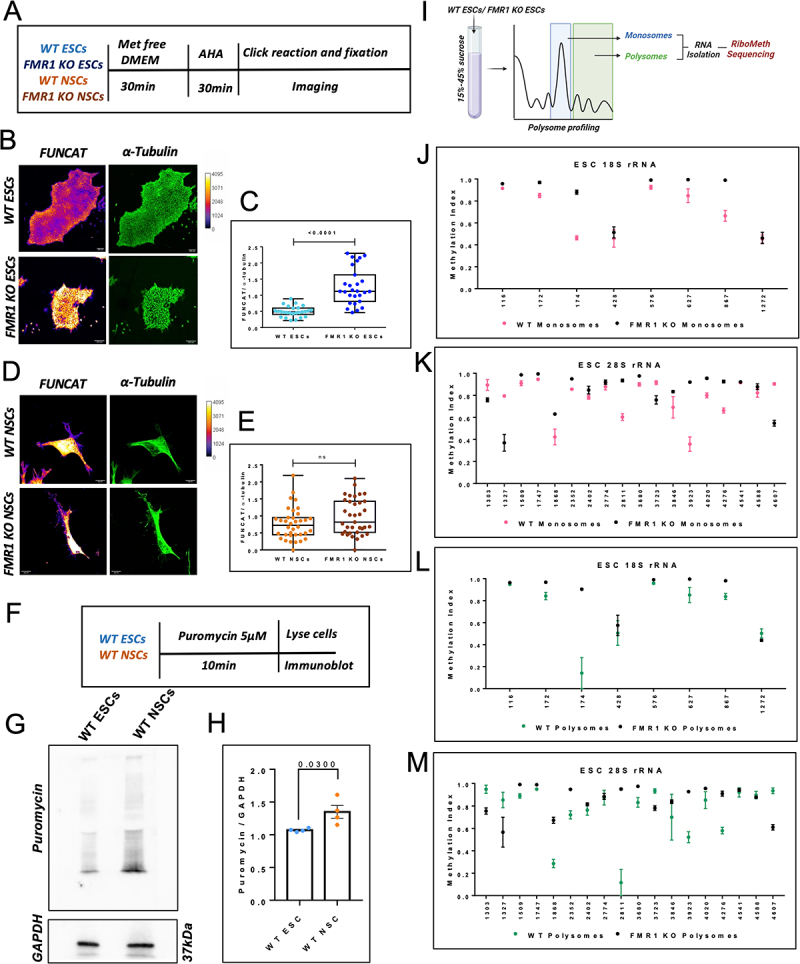
Altered 2’O-methylation affects translation in ESCs and NSCs.

Cell state transitions are controlled by changes in global protein synthesis. To capture changes in global protein synthesis in the absence of FMRP along the differentiation of ESC to NSC, we made use of a non-canonical amino acid tagging system called FUNCAT [20]. The rate of production of newly synthesized proteins was measured in WT and FMR1 KO ESCs and NSCs through the quantification of the FUNCAT signal, which was normalized to endogenous α-tubulin protein levels in each condition (Figure 4(A)). We observed that isolated ESCs lose their pluripotency signal upon separation from the ESC colony. Hence, we quantified the total FUNCAT and α-tubulin signal from whole ESC colonies and not from individual cells, as we did in the case of NSCs. Our results indicate that the absence of FMRP caused a significant upregulation of global protein synthesis in ESCs compared to the WT condition (Figure 4(B,C)). Interestingly, we did not capture this trend in the differentiated NSCs (Figure 4(D,E)). There was no significant difference observed in the rates of translation between WT and FMR1 KO NSCs (Figure 4(D,E)). This finding indicates that FMRP might have a prominent role in regulating translation at early developmental stages as opposed to intermediate stages of differentiation. To confirm our observations, we also measured rates of global protein synthesis in WT and FMR1 KO ESCs by quantifying the levels of puromycin incorporation between the two conditions (Figure 4(F)). We observe a significant increase in the levels of puromycin-labelled proteins in the absence of FMRP (FMR1 KO ESCs), indicating an overall increase in translation (Figure S4A-S4D). Since we hypothesize that increased 2’O-methylation on rRNA could result in increased rates of translation, and WT NSCs have hypermethylated rRNA residues compared to WT ESC rRNA [1,21–23], we examined the rates of protein synthesis between WT ESCs and WT NSCs by measuring the levels of puromycin-labelled proteins. We did not measure this parameter through FUNCAT since it is not possible to compare the FUNCAT intensity between ESC colonies and individual NSCs. Our data shows that there is a significant increase in puromycin incorporation in WT NSCs compared to WT ESCs, indicating that overall protein synthesis increases as cells differentiate from ESCs to NSCs (Figure 4(G-H) and Figure S4E-F).

A. Schematic describing FUNCAT assay for the measurement of de-novo protein synthesis in WT and FMR1 KO ESCs and NSCs. B. Representative images for FUNCAT and α-Tubulin fluorescent intensities in WT and FMR1 KO ESCs. (Scale bar—100 µm). C. Box plot representing the quantification of the FUNCAT intensity normalized to α-Tubulin intensity for each colony of WT ESC and FMR1 KO ESC. The box extends from the 25th to the 75th percentile, with the middlemost line representing the median of the dataset. Whiskers range from minimum to maximum data points. Unpaired t-test. *n* = 30–35 colonies from 3 independent experiments. D. Representative images for FUNCAT and α-Tubulin intensities in WT and FMR1 KO NSCs. (Scale bar—50 µm). E. Box plot representing the quantification of the FUNCAT intensity normalized to α-Tubulin intensity for each cell of WT and FMR1 KO NSC. The box extends from the 25th to the 75th percentile, with the middlemost line representing the median of the dataset. Whiskers range from minimum to maximum data points. Unpaired t-test. *n* = 35–36 cells from 3 independent experiments. F. Schematic describing Puromycin labelling of newly synthesized proteins to measure rates of translation in WT ESCs and WT NSCs. G. Immunoblot indicating an increase in Puromycin incorporation (5 µM for 10 min at 37ºC) in WT NSCs compared to WT ESCs. 50 µg of total protein was loaded per sample. H. Quantification of Puromycin incorporation in WT ESCs and WT NSCs. Data is normalized to respective GAPDH levels. Mean ± SEM from *n* = 4 independent experiments. I. Schematic for RiboMeth-Seq from WT ESCs input, monosome, and polysome fractions after polysome profiling. WT ESCs were subjected to sucrose density gradient centrifugation on a 15–45% sucrose gradient. RNA was extracted from WT ESCs input, monosome, and polysome fractions and processed for RiboMeth Sequencing. J. Methylation indices of the sites on 18S rRNA in WT ESC monosomes and FMR1 KO ESC monosomes. The x-axis represents the methylation positions on 18S rRNA, and the y-axis represents the Methylation Index. Data is represented as Mean ± SEM, *n* = 3. K. Methylation indices of the sites on 28S rRNA in WT ESC monosomes and FMR1 KO ESC monosomes. The x-axis represents the methylation positions on 28S rRNA, and the y-axis represents the Methylation Index. Data represented as Mean ± SEM, *n* = 3. L. Methylation indices of selected sites (MI > 0.90) on 18S rRNA in ESC WT and FMR1 KO polysomes. The x-axis represents the methylation position on 18S rRNA, and the y-axis represents the Methylation Index, Data is represented as Mean ± SEM, *n* = 3. M. Methylation indices of selected sites (MI > 0.90) on 28S rRNA in ESC WT and FMR1 KO polysomes. The x-axis represents the respective methylation position on 28S rRNA, and the y-axis represents the Methylation Index. Data is represented as Mean ± SEM, *n* = 3.

From our previous study, we know that FMRP regulates translation by affecting the epitranscriptome of the ribosome [11]. We aimed to understand the effect of FMRP on the 2’O-methylation of translating monosomes and polysomes. For this, total cell lysate from WT and FMR1 KO ESCs was loaded on a linear sucrose density gradient, and components were separated based on density through ultracentrifugation. We collected RNA from the pools of monosomes and polysomes from WT and FMR1 KO conditions and subjected the RNA to RiboMeth-Seq (Figure 4(I)) . Our data shows that there are more hypomethylated sites in the 28S rRNA compared to 18S rRNA in both monosome and polysome populations, and the absence of FMRP results in the hypermethylation of a majority of these sites in both the ribosomal populations (Figure 4(J-M) and S4G-L).

## Discussion

**Figure 5.**
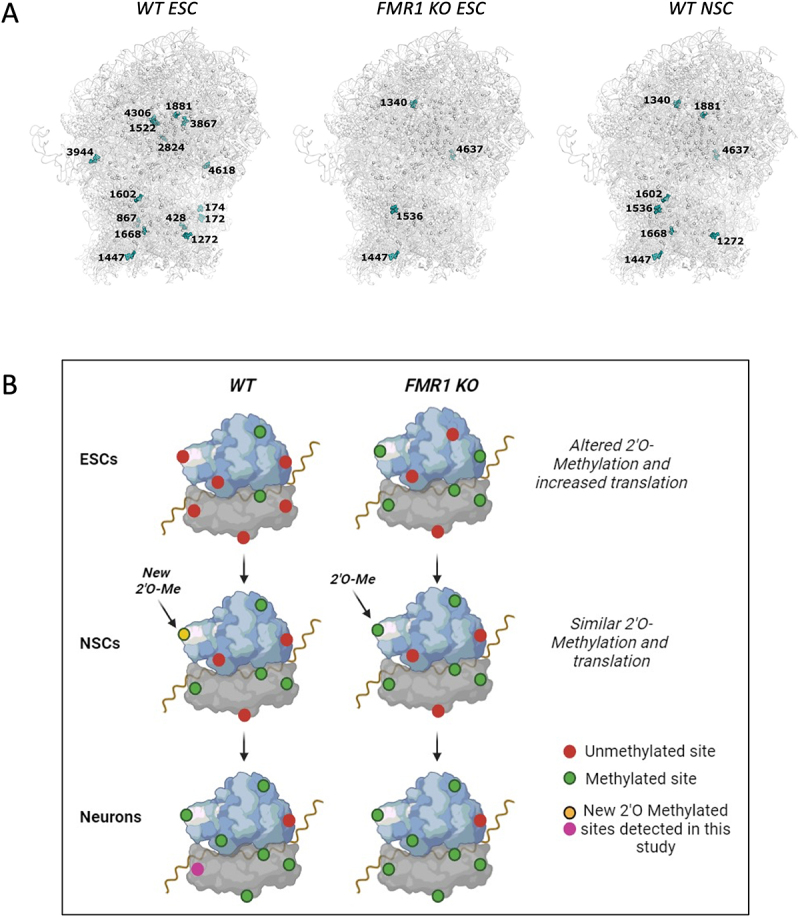
Altering 2’O-Methylations on ribosomes during neuronal differentiation.

Cellular differentiation is an event where a state of specialization is achieved to facilitate a unique cellular function. A combination of various processes, such as transcription, epigenetic changes, epitranscriptomic changes, protein synthesis, and cell signalling, contributes to this process. Our study focuses on heterogeneous ribosomes generated from epitranscriptome changes to the rRNA, which provide an important layer of complexity to protein synthesis during neuronal differentiation of pluripotent embryonic stem cells. Ribosomal RNA heterogeneity is generated primarily through altered sequence or epitranscriptomic modification of the rRNA [4,7,8,24,25]. Our study shows a distinct pattern of 2’O-methylation on both 18S and 28S rRNA in human ESCs. We observe that a subset of these sites on rRNA is hypomethylated in the stem cell state. However, these sites get further methylated when the cells are differentiated along the neuronal lineage. These results are in line with the findings of another recently published manuscript, which offers confidence to our observed phenomena [4]. As cells achieve their post-mitotic fate, we observe the highest number of completely methylated sites. In other words, there is more 2’O-hypomethylation of rRNAs in ESCs, which reduces as ESCs are differentiated into NSCs and neurons (Figures 1(B,C), and 5(B)). This is a very surprising result since neurons are highly polarized cells that require elaborate compartmentalized and activity-mediated protein synthesis. Therefore, we expected a higher level of heterogeneous ribosomes in them. On the contrary, our results indicate that the highest level of rRNA 2’O-hypomethylation is in ESCs, and relatively reduced 2’O-methylation is in neurons. While considering our results, it is important to note that our RMS was performed with lysates from whole neurons and not from specific compartments. Neurons show localized protein translation at the synapses, tightly regulated by synaptic activity [26–28]. Hence, it is possible that there could exist a higher level of hypomethylated ribosomes within these compartments. Broadly, our data suggests that ESCs possess higher rRNA hypomethylation, which we correlate with the pluripotent state of the cell.

A. Front view of 2’O-methylation sites on WT ESC, FMR1 KO ESC, and WT NSC ribosomes. Hypomethylated sites are indicated in blue (MI < 0.75). B. Graphical abstract indicating altered patterns of 2’O-Methylation on rRNA across neuronal differentiation and in the absence of FMRP.

We observe a maximum number of hypomethylated sites in ESCs, indicating a very high number of partially methylated ribosomes. This seemingly counterintuitive finding becomes logical once we carefully consider the pluripotent nature of ESCs. Translation rate is presumably low in ESCs and is thought to go up as they differentiate [1]. Cellular differentiation is a highly dynamic process that occurs in response to various intrinsic and extrinsic cues. This requires quick proteomic remodelling, which is largely determined by translation regulation. It is essential for ESCs to respond to these cues either by enhancing transcription or by priming the existing ribosomes [15,16]. Here, we argue that a high level of hypomethylated ribosomes provides ESCs with such potential. The high level of hypomethylated rRNA in ESCs suggests that there are large numbers of different ribosomal pools, each having its distinct pattern of methylation (Figure 4(J-M)). We argue that since ESCs are primed to differentiate into multiple germ layers, the system is equipped to translate different pools of mRNAs when required. In other words, we propose that pluripotency in ESCs is maintained because of the multiple pools of heterogeneous ribosomes. Once differentiation is initiated, rRNA gets hypermethylated, and the cells begin to produce a more homogenous population of ribosomes.

Previously, we have shown that FMRP associates with a specific set of C/D box snoRNAs in ESCs, and this can lead to the generation of specific rRNA methylation patterns and thus heterogeneous ribosomes [11]. We also indicate that this association is primarily in the nucleus of ESCs, as shown earlier, although the biochemical nature of this interaction is unknown [11]. Our current data shows that the steady-state expression of specific snoRNAs is unaltered between ESCs and NSCs. Further, the expression of specific snoRNAs is not affected in the absence of FMRP (Figure 3(B) and Figure S3A). This finding was anticipated as snoRNAs are very abundant yet essential for the process of ribosome biogenesis [29]. However, this result did not explain the differences in 2’O-methylation that we captured between ESCs and NSCs. Further, this also did not explain the trend of hypermethylation we observed in conditions where FMRP is absent. We hypothesize that the differential rRNA methylation observed between cell states could, in part, be explained by the differential interaction of FMRP with its target C/D Box snoRNAs. Our earlier work has shown that the interaction of FMRP with these snoRNAs is similar across different ESC lines, such as Shef4 ESCs and H9 ESCs [1,2]. In the current study, we find that the extent of FMRP – snoRNA interaction is significantly reduced from ESCs to NSCs (Figure 3(C)), suggesting that FMRP binding is strongest in ESCs. This higher sequestration of snoRNAs in ESCs may reduce their availability to guide methylation at rRNA target sites (Figure 3(D)), contributing to the higher number of hypomethylated sites detected in ESC ribosomes. Conversely, in NSCs, the reduced sequestration of snoRNAs could increase their availability, leading to more methylation at the corresponding rRNA sites (Figures 3(E) and 5(B)). A similar pattern is observed in FMR1 KO ESCs, where the absence of FMRP results in higher availability of target snoRNAs and hypermethylation of rRNA (Figures 3(E) and 5(B)). While these findings are consistent with a model in which FMRP-dependent snoRNA sequestration modulates rRNA methylation, we acknowledge that this mechanism likely accounts for only a subset of the observed changes. Broader shifts in rRNA 2’-O-methylation during neuronal differentiation and upon FMR1 loss are likely influenced by additional regulatory layers, such as changes in snoRNP expression, localization, and activity, as well as global alterations in ribosome biogenesis and cell state.

Since there is mounting evidence to link epitranscriptomic changes in rRNA to altered translation [30–34], we decided to test the effect of altered rRNA 2’O-methylation on global protein synthesis in the ESCs and NSCs in the presence or absence of FMRP. FMRP is a regulator of translation; however, it is predominantly known to inhibit translation through the stalling of ribosomes [35,36]. Our analysis indicates that FMR1 KO ESCs show a significant upregulation of overall protein synthesis compared to WT H9 ESCs (Figures 4(B,C)). This phenomenon is only evident at the ESC stage. There was no difference in the global protein synthesis between WT-NSCs and FMR1 KO NSCs. Interestingly, the correlating observation of rRNA hypermethylation in some sites to increased protein synthesis was captured when we compared ESCs with their differentiated neuronal precursor forms (Figure 4(G,H)). Since protein expression during cellular differentiation is largely controlled by ribosomal function, this regulation is likely determined by cell-state-specific heterogeneous ribosomes. Currently, there are only a limited number of studies showing the consequence of a 2’O-methylated rRNA residues on protein synthesis [25,37,38]. However, it appears that rRNA hypermethylation correlates with increased translation and hypomethylation reduces translation, which requires direct validation through extensive experimentation.

In addition, our general observation shows that 28S rRNA possesses the maximum number of hypomethylated residues (40 significantly altered sites out of 64 sites) in comparison to the 18S rRNA (21 significantly altered sites out of 39 sites). This result is in line with the concept that the rRNA of the ribosomal small subunit is less variable compared to the large subunit [39, 40]. Also, the absence of FMRP causes hypermethylation of a handful of sites across 18S and 28S rRNA in both the ribosomal populations (Figure 5(B)). This implies that the occurrence of heterogeneous ribosomes and their alteration due to the loss of FMRP is only significant at the early stages of embryonic development (Figure 5(B)). Further, the importance of FMRP in regulating ribosome biogenesis might be particularly relevant at the ESC state, while FMRP might adopt alternate roles in the NSCs and neurons to regulate protein synthesis.

The scope of this study is specifically to explore a potential link between FMRP and translational regulation through modulation of ribosomal 2′O-methylation during neuronal differentiation. We acknowledge that FMRP has multiple roles, including mRNA binding, ribosome association, and regulation of translation elongation; however, its mRNA-binding functions have been extensively characterized in previous studies and are beyond the focus of the present work. Instead, here we centre our investigation on FMRP’s potential to influence ribosome heterogeneity via its interaction with C/D box snoRNAs. Detailed mechanistic aspects of FMRP’s interaction with ribosomes and snoRNAs have been discussed in our earlier publications from the lab, which provide additional context for the molecular framework underlying the observations reported here [41,42].

In summary, our study identifies FMRP-dependent ribosome heterogeneity in ESCs and suggests a potential link to translational regulation, as supported by our translation assays and snoRNA interaction analyses. This will be one of the few reports to show how RNA-binding proteins like FMRP contribute to differential 2’O-methylation in the pluripotent stem cells and their differentiation [4,43–45]. Our data suggest that the key methylation positions vary between WT and FMR1 KO ribosomes and along differentiation around the PTC (Peptidyl Transferase Center) (Figure 5(A)), and studying them in detail would open up many interesting avenues to understand 2’O-methylation-dependent translation regulation. Further, our study also shows how distinct 2’O-methylation patterns on rRNA can be used as indicators of specific cell states during differentiation and development.

## Materials and methods

### Ethics statement

All the human stem cell work was carried out as per approval from the Institutional Human Ethics Committee and Institutional Biosafety Committee at InStem, Bengaluru, India, and the Centre for Brain Research, Indian Institute of Science Campus, Bangalore, India.

### Embryonic stem cell culture

Human embryonic stem cells (H9 ESCs) and FMR1 knockout ESCs were cultured as per the protocol outlined in D’Souza et al [11]. ESCs were grown on Matrigel-coated plates in mTeSR1 medium under standard conditions (37°C, 5% CO₂). For passaging, cells were treated with an enzyme mixture containing Collagenase type IV, KOSR, Trypsin, and CaCl₂ dissolved in PBS (without Ca^2 +^ or Mg^2 +^, pH 7.2). For immunostaining, H9 ESC colonies were plated on Matrigel-coated glass coverslips and cultured as described [11]. Differentiation of H9 ESCs into neural precursor cells (NPCs) was achieved using Neural Induction Medium over 14 days, based on the method from Shi et al., which induces iPSCs to differentiate into forebrain glutamatergic neurons [19]. The Neurobasal Media (NBM) used for differentiation comprised 50% DMEM F-12, 50% Neurobasal, PenStrep, Glutamax, N2, and B27 without Vitamin A. Upon reaching 70–80% confluence, ESCs were exposed to Neural Induction Media (NIM), which includes NBM along with the TGFβ inhibitor SB431542 (10 µM) and the BMP inhibitor LDN193189 (0.1 µM). Neural induction continued for 12–15 days, with daily media changes, until a uniform neuroepithelial layer formed. After induction, cells were dissociated with Accutase, centrifuged, and plated overnight in NIM with 10 µM ROCK inhibitor on poly-L-ornithine/laminin-coated dishes. The NSCs were maintained in Neural Expansion Media (NEM) composed of NBM with FGF (10 ng/ml) and EGF (10 ng/ml). For neuronal maturation and terminal differentiation, NSCs were plated at a density of 25,000–35,000 cells/cm^2^ in Neural Maturation Media (NMM), containing NBM with BDNF (20 ng/ml), GDNF (10 ng/ml), L-Ascorbic Acid (200 µM), and db-cAMP (50 µM). Neurons were allowed to mature for 4–5 weeks, with media changes every 4–5 days.

### Characterization of stem cells

ESCs, NSCs, and neurons were characterized by immunofluorescence using a previously established protocol [46]. In brief, cells were fixed with 4% PFA for 15 minutes, followed by 1X PBS wash and permeabilization with 0.3% Triton X-100 (made in TBS_50_ −50mMTris and 150 mM NaCl) for 10 minutes. This was followed by 1 hour blocking with 2% BSA and 2% FBS prepared in TBS_50_T (with 0.1% Triton X-100). They were incubated with the primary antibody (prepared in blocking buffer) overnight at 4°C. This was followed by 3 washes with TSB_50_T and 1-hour incubation with the secondary antibody (prepared in blocking buffer) at room temperature. After 3 washes with TBS_50_T, the cells were mounted with Mowiol.

### Metabolic labelling

We labelled cells metabolically following an established protocol [46]. ESCs and NSCs were incubated in methionine-free DMEM (Thermo# 21013024) for 30 minutes, then treated with azidohomoalanine (AHA; 1 μM (Thermo# 21013024)) for another 30 minutes. After fixation with 4% PFA, cells were permeabilized, blocked, and labelled with Alexa Fluor 555–alkyne (Thermo #A20013,) via click chemistry (Thermo#C10269) to detect newly synthesized proteins. Immunostaining for α-tubulin (ESCs and NSCs) was performed for cell identification, and coverslips were mounted using Mowiol® 4–88 media (Sigma #81381).

### Imaging

Mounted coverslips were imaged using an Olympus FV3000 confocal laser scanning microscope with a 20X objective. The pinhole was set to 1 Airy Unit, and the optical zoom to 2X to meet Nyquist sampling in the XY direction. Z-stacks were collected with a 1 μm step size (~8–10 slices) to capture light from above and below the focal plane. NSCs and ESC colonies were identified via the α-tubulin channel for FUNCAT. Image analysis was performed in ImageJ, with maximum intensity projections used to quantify mean fluorescence intensities. Regions of interest (ROIs) were drawn using the α-tubulin channel, and FUNCAT fluorescence intensities were normalized to α-tubulin levels. FUNCAT intensities were measured from individual Neural stem cells and whole ESC colonies. The results are shown as box plots, with boxes representing the 25th to 75th percentiles, medians, and whiskers extending from the minimum to maximum values.

### Linear sucrose density centrifugation

Polysome assay was done from WT and FMR1 KO ESC lysate as described previously [47]. In brief, cell lysate was separated on a 15%–45% linear sucrose gradient in the presence of 0.1 mg/ml Cycloheximide (CHX) (Sigma #C7698-5 G) and Phosphatase inhibitor (Roche #4906837001) by centrifugation at 39,000 rpm in the SW41 rotor for 90 min. The sample was fractionated into 12 1.0 mL fractions with continuous UV absorbance measurement (A254). Fractions were pooled as monosomes (F4 and F5) and polysomes (F6–12) according to ribosomal subunit distribution based on the peaks. RNA was isolated from the pooled fractions and subjected to RiboMeth-Seq and RTL-P.

### snoRNA quantification

CDNA of snoRNA was prepared using reverse primers specific to individual snoRNA candidates [11]. CDNA was amplified using SYBR premix by qPCR. Arbitrary copy numbers were calculated from a standard curve drawn from Ct values obtained from serial dilutions of cDNA for snoRNA candidate HBII99. Copy numbers for various snoRNA candidates were obtained using the equation generated from the standard curve.

### Immunoprecipitation

ESCs and NSCs were lysed in 1% NP40 containing lysis buffer (20 mM Tris-HCl, pH 7.5, 150 mM NaCl, 5 mM MgCl2 with protease and RNase inhibitors) and spun at 18,000 rcf (12500 rpm) for 20 minutes at 4°C. Precleared supernatant was used for immunoprecipitation with Protein G Dynabeads. 5 μg of anti-FMRP antibody (Sigma #F4055) was coupled to the Protein G Dynabeads. Lysates were incubated with antibody-conjugated beads for 1 h at RT, following which RNA was isolated using Trizol.

### Immunoblotting

Lysates from WT and FMR1 KO cells were analysed by western blot for FMRP expression [11]. Denatured lysates were run on 10% resolving and 5% stacking acrylamide gels, followed by overnight transfer onto a PVDF membrane. The membrane was blocked for 1 hour at room temperature using 5% Blotto in TBST (TBS with 0.1% Tween-20). After blocking, the blots were incubated with the primary antibody in blocking buffer (Sigma #F4055) for 3 hours at room temperature, followed by HRP-tagged secondary antibody incubation for 1 hour. Blots were washed three times with TBST after both antibody incubations. Protein detection was performed using chemiluminescence to visualize the HRP-tagged proteins.

### RiboMeth-Seq and analysis

RNA for RiboMeth-Seq was processed as per our previous publication [11]. In brief, 2 μg of total RNA extracted from WT and FMR1 KO ESCs, NSCs, neurons, and ribosomal fractions was used for library preparation. RNA was hydrolysed with alkaline Tris buffer (pH 10) at 95°C for 5 minutes and ethanol precipitated. Isolated RNA was run on a 12% TBE PAGE gel, and a band corresponding to 30–50 bp was excised. Sequencing libraries were prepared using the TruSeq small RNA library preparation Kit from Illumina and were sequenced on the HiSeq 2500 platform. FastQC (v0.11.5) was used to assess the quality of the 50bp reads across all the samples. Adapter sequences (TGGAATTCTCGGGTGCCAAGG) were trimmed using Cutadapt (v2.10). The trimmed reads were aligned to the reference rRNA sequences (ENST00000606783–18S rRNA & ENST00000607521–28S rRNA) using bowtie (v1.1.2) with default parameters in the end-to-end mode. The alignment files were sorted and indexed using samtools (v1.7), which were then used for counting the number of 5’and 3’ read-ends that were mapped to each position on the reference rRNA using bedtools (v2.25.0). The 5’counts were shifted up by one position and combined with the 3’ counts to ascertain the methylated positions in the reference sequence. Further, RiboMeth-Seq scores were calculated for all the known methylated positions (64 from 28S rRNA and 42 from 18S rRNA) using custom bash and awk scripts. Heatmaps of the score C from the various samples were plotted in R using the package ‘pheatmap’. In our comparisons, the term ‘hypomethylation’ is defined as a site’s MI score is ≤ 0.9. We used WT ESCs as a baseline, and significant t-test values indicate hypomethylation when comparing their scores in the differentiated or FXS condition.

### RTL-P (reverse transcription at low-dNTP concentration followed by PCR)

2 ng of sample RNA was used for cDNA preparation using reverse primers (10 μM) specific to methylation sites under high dNTP (10 mM) and low dNTP (1 nM) concentrations. For real-time PCR, we adopted a method from *Dong et al., 2012*[17]. We have used two forward primers for a methylation site; one up-stream (P1) and one down-stream (P2) from the methylation site, along with a common reverse primer (P3). Amplification with these sets of primers would yield one product over the methylation site which will be the longer product and another will be within the methylation site and would yield a shorter product. The extent of methylation for a given site is calculated as a methylation score as previously described in *D’Souza et al, 2019*[11].

### Puromycin labelling

ESCs and NSCs were incubated with 5 µM Puromycin (Sigma # P8833-25 MG) for 10 minutes and were lysed in buffer (20 mm Tris-HCl, 100 mm KCl, 5 mm MgCl_2_, 1% NP40, 1 mm DTT, 1× protease inhibitor cocktail, and 1× phosphatase inhibitor). The protein levels were quantified from precleared lysates using the BCA method (Thermo # 23227). 50 µg of total protein was loaded for all samples on a 10% SDS polyacrylamide gel. Immunoblots were stained with ponceau to ensure the successful transfer of proteins. The blots were blocked in 5% BSA made in 1× TBST. The blots were incubated in puromycin antibody (Sigma # MABE343; 1:10000) for 3 hours at RT, followed by anti-mouse HRP antibody (Sigma # A9044; 1:10000) for 1 hour at room temperature. The same immunoblots were stripped of the puromycin antibody (62.5 mM Tris Buffer (pH 6.8), 2% SDS, 0.7% β-Mercaptoethanol). This was followed by incubation with GAPDH (Cell Signaling Technologies #2118S; 1:5000) for 1 hour at room temperature and anti-rabbit HRP antibody (Sigma # A0545; 1:10000) for 1 hour at room temperature. The puromycin signal was normalized to the GAPDH levels.

### Statistical analysis

All statistical analyses were performed using GraphPad Prism software. Data normality was assessed with the Kolmogorov-Smirnov test. Parametric tests were used for experiments with fewer than 5 data points. In-vitro and polysome experiment data were presented as mean ± SEM, while FUNCAT data were shown as box-and-whisker plots with individual data points [42]. Statistical significance for two-group comparisons was calculated using the unpaired Student’s t-test (two-tailed, equal variance). For multiple group comparisons, one-way ANOVA was followed by Tukey’s, Bonferroni’s or Dunnett’s multiple comparison tests. The unpaired Student’s t-test was also used for snoRNA qPCR and puromycin incorporation assays.

## Supplementary Material

Supplemental Material

## Data Availability

The data that support the findings of this study are available in NCBI Genbank at https://dataview.ncbi.nlm.nih.gov/object/PRJNA1129659?reviewer=oe0h4lu4haoqgg16132a2dt6f2, BioProject Accession: PRJNA1129659.
