# Supplementary figures and images for: Altering rRNA 2’O-methylation pattern during neuronal differentiation is regulated by FMRP
Source: RNA Biol. 2025 Oct 3;22(1):1–22. doi: 10.1080/15476286.2025.2563986 (PMC12498540; doi:10.1080/15476286.2025.2563986)

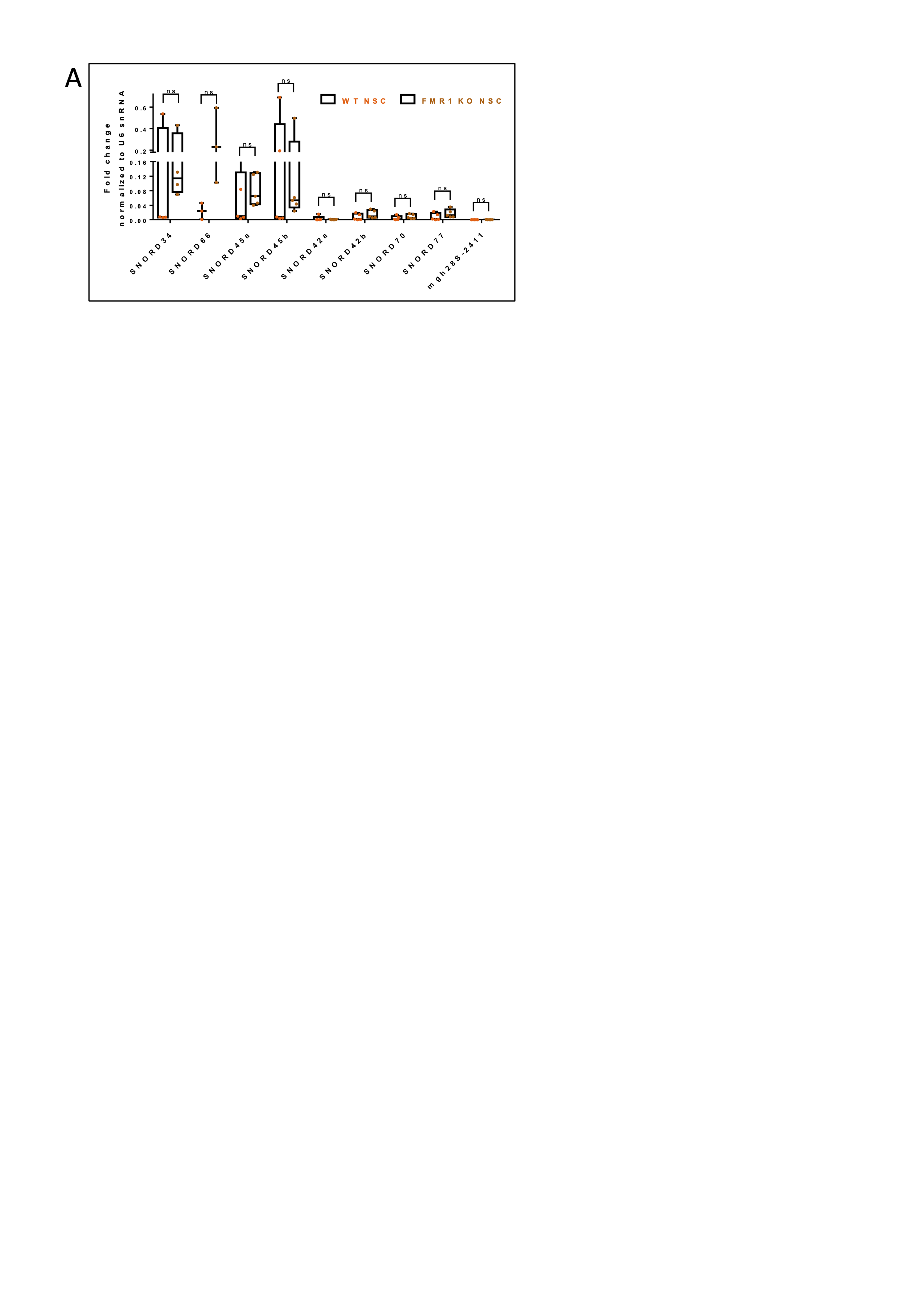

Supplement: Supplemental Material [file KRNB_A_2563986_SM8848.zip › Figure 3 Sup.tiff]

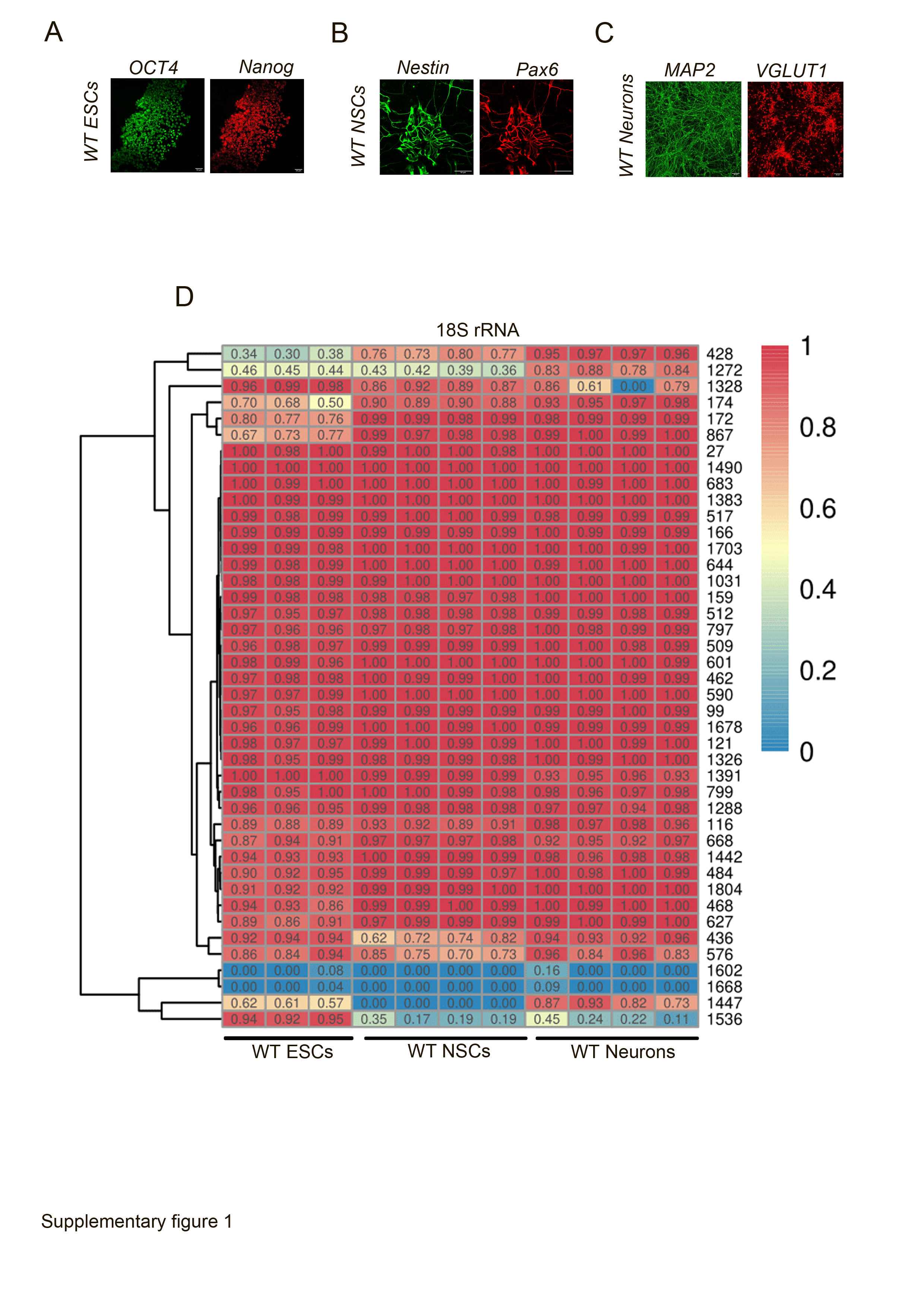

Supplement: Supplemental Material [file KRNB_A_2563986_SM8848.zip › Figure 1 Supp.tiff]
